# RAS mutant transverse colon cancer with multiple liver metastases achieving long-term disease-free survival with postoperative maintenance therapy with aflibercept + FOLFIRI and four repeated radical resections: a case report

**DOI:** 10.1186/s40792-024-02033-2

**Published:** 2024-10-08

**Authors:** Yasushi Tanaka, Ryota Nakanishi, Shota Sato, Akihiko Otake, Keiichiro Ryujin, Shinichiro Ikeda, Yuho Ebata, Tomoya Harima, Keita Natsugoe, Takayuki Yoshiyama, Yuki Shin, Tetsuro Kawazoe, Kensuke Kudo, Yoko Zaitsu, Yuichi Hisamatsu, Koji Ando, Yuichiro Nakashima, Shinji Itoh, Eiji Oki, Yoshinao Oda, Tomoharu Yoshizumi

**Affiliations:** 1 Department of Surgery and Science, Kyushu University, 3-1-1, Maidashi, Higashi-Ku, Fukuoka, 812-8582 Japan; 2 Department of Anatomic Pathology, Pathological Sciences, Graduate School of Medical Sciences, Kyushu University, 3-1-1, Maidashi, Higashi-Ku, Fukuoka, 812-8582 Japan

**Keywords:** Colorectal liver metastasis, RAS mutant, Aflibercept, Metachronous metastases, Multidisciplinary treatment

## Abstract

**Background:**

Management of patients with colorectal liver metastases (CRLMs) requires a multidisciplinary approach. For patients with progression of RAS mutant tumors, the choice of angiogenesis inhibitors can be controversial. Here, we report a patient with RAS mutant CRLMs achieving long-term disease-free survival with repeated R0 resections and perioperative treatment, especially aflibercept + FOLFIRI (5-fluorouracil, levofolinate, irinotecan), which may have prevented long-term recurrence.

**Case presentation:**

The patient was a 37 year-old woman diagnosed with RAS mutant transverse colon cancer with 19 LMs. As the metastases were limited to the liver, we introduced systemic chemotherapy aiming at conversion surgery. After six cycles of bevacizumab + FOLFOXIRI (5-fluorouracil, levofolinate, oxaliplatin, irinotecan), we performed partial hepatectomy for all LMs, and left hemicolectomy for the primary tumor after another four cycles of bevacizumab + FOLFIRI. Three months after surgery, the patient presented with massive ovarian metastases with carcinomatous ascites. We conducted bilateral oophorectomy, and initiated aflibercept + FOLFIRI therapy considering the possibility of resistance to bevacizumab. The patient was recurrence-free for 2 years during aflibercept + FOLFIRI treatment. After its discontinuation, two distant metastases developed. Both were resectable and the patient achieved recurrence-free survival of 2 years and 3 months after the last operation (6 years since initiation of treatment), without additional chemotherapy.

**Conclusions:**

We believe that multidisciplinary treatment aimed at complete resection could lead to long-term survival even in patients with repeated recurrence of CRLMs. Aflibercept + FOLFIRI could be effective in controlling metastasis of RAS mutant colon cancer even after treatment with bevacizumab.

## Introduction

Colorectal cancer (CRC) is the second most common cancer in Europe and the second leading cause of cancer-related mortality worldwide [1]. At the time of initial diagnosis, up to 20% of patients with CRC have liver metastases (LMs), which are associated with poor tumor biology and prognosis [2]. Especially in the case of multiple LMs in both lobes, the management of patients with colorectal LMs (CRLMs) requires a complex, multidisciplinary approach involving colorectal surgery, hepatic resection, and chemotherapy at appropriate intervals. Bevacizumab + FOLFOXIRI (5-fluorouracil, levofolinate, oxaliplatin, irinotecan) has recently become the preferred treatment for multiple LMs because of patients’ high response rate and prolonged survival [3, 4]. However, the choice of drugs following disease progression after bevacizumab + FOLFOXIRI remains controversial especially in case of RAS mutant. FOLFIRI (5-fluorouracil, levofolinate, irinotecan) + aflibercept or ramucirumab can be beneficial [5–7], but the evidence on which drugs to use after bevacizumab + FOLFOXIRI needs to be clarified.

Here, we present a patient with RAS mutant transverse colon cancer with synchronous multiple LMs. We used bevacizumab + FOLOFOXIRI as first-line therapy in combination with surgery. Long-term survival was achieved after recurrence with repeated resection and continued treatment with aflibercept + FOLFIRI.

## Case report

**Fig. 1:**
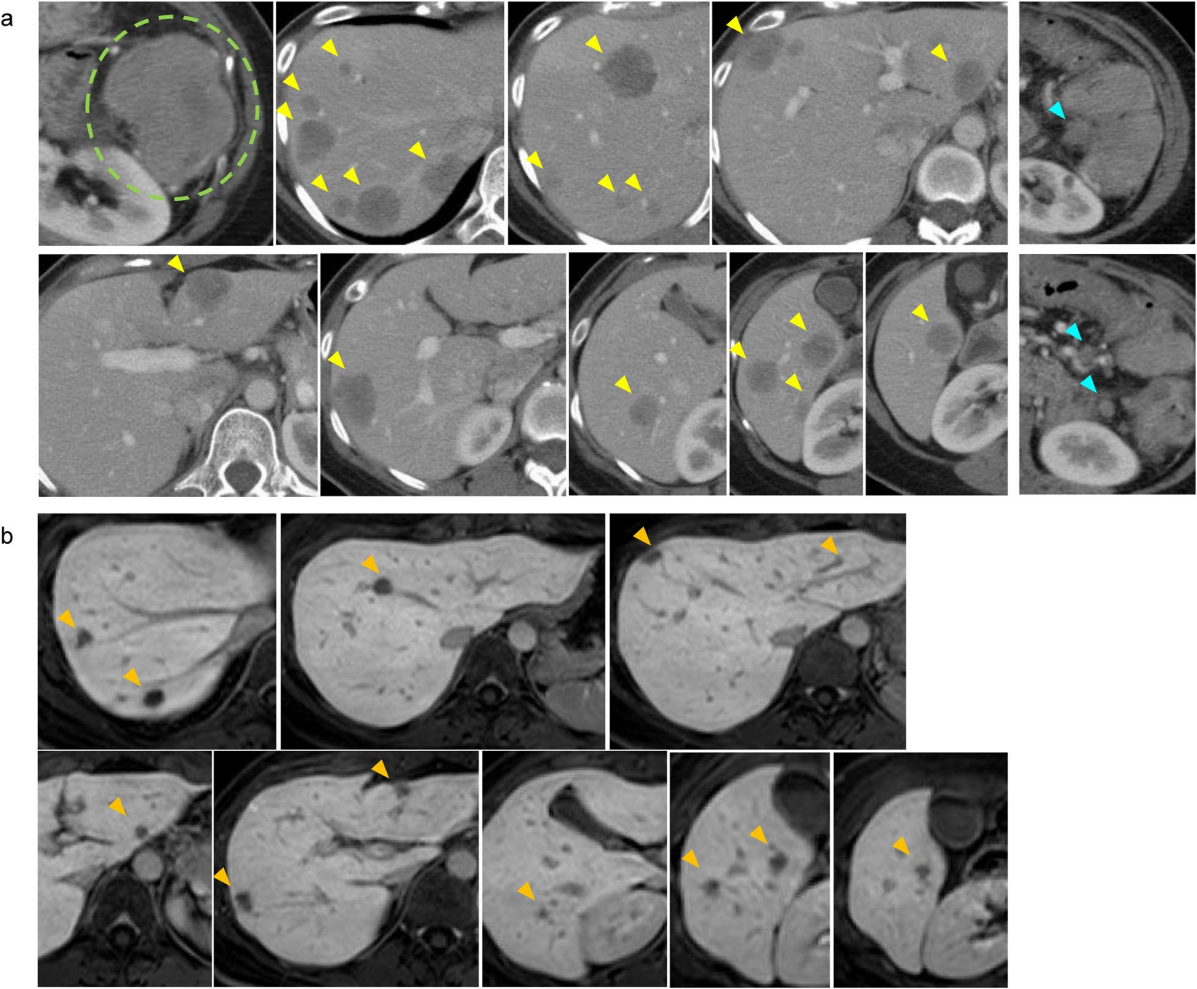
**a** Computed tomography at the initial examination showed thickened walls of the transverse colon at the splenic flexure (green circle), swollen lymph nodes in the mesocolon near the tumor (blue allows), and 19 multiple solid low-density masses in the liver (yellow allows). **b** Magnetic resonance imaging after chemotherapy showed shrinkage of all liver metastases in both lobes (orange arrows)

The patient was a 37 year-old woman, 17 weeks pregnant. She had abdominal pain and diarrhea for 3 weeks. Colonoscopy revealed severe stenosis caused by a tumor at the splenic flexure. Histological examination of the tumor revealed well- to moderately-differentiated adenocarcinoma. The cancer genotype was MSS, RAS mutant (KRAS G12V) and BRAF wild type. She did not have any family history of cancer. Ultrasound and magnetic resonance imaging showed multiple hepatic masses.6 After she had a transverse colostomy and an abortion at a previous hospital, she was referred to our institution for further examination and treatment. Biochemical examination revealed that carcinoembryonic antigen level was elevated to 1402 ng/mL and tumor-associated carbohydrate antigen 19–9 to 693.6 U/mL. Abdominal contrast-enhanced computed tomography (CT) showed thickened walls of the transverse colon at the splenic flexure, three swollen lymph nodes in the mesocolon near the tumor, and 19 multiple solid low-density masses in the liver (Fig. 1a). Positron emission tomography/CT showed moderately intense accumulation of 18F-fluorodeoxyglucose in tumors in the colon and liver, and swollen lymph nodes. No other distant metastases were detected. According to these examinations, the final diagnosis was transverse colon cancer and synchronous 19 liver metastases [cT4aN1bM1a(H3), c-Stage IVa].

**Fig. 2:**
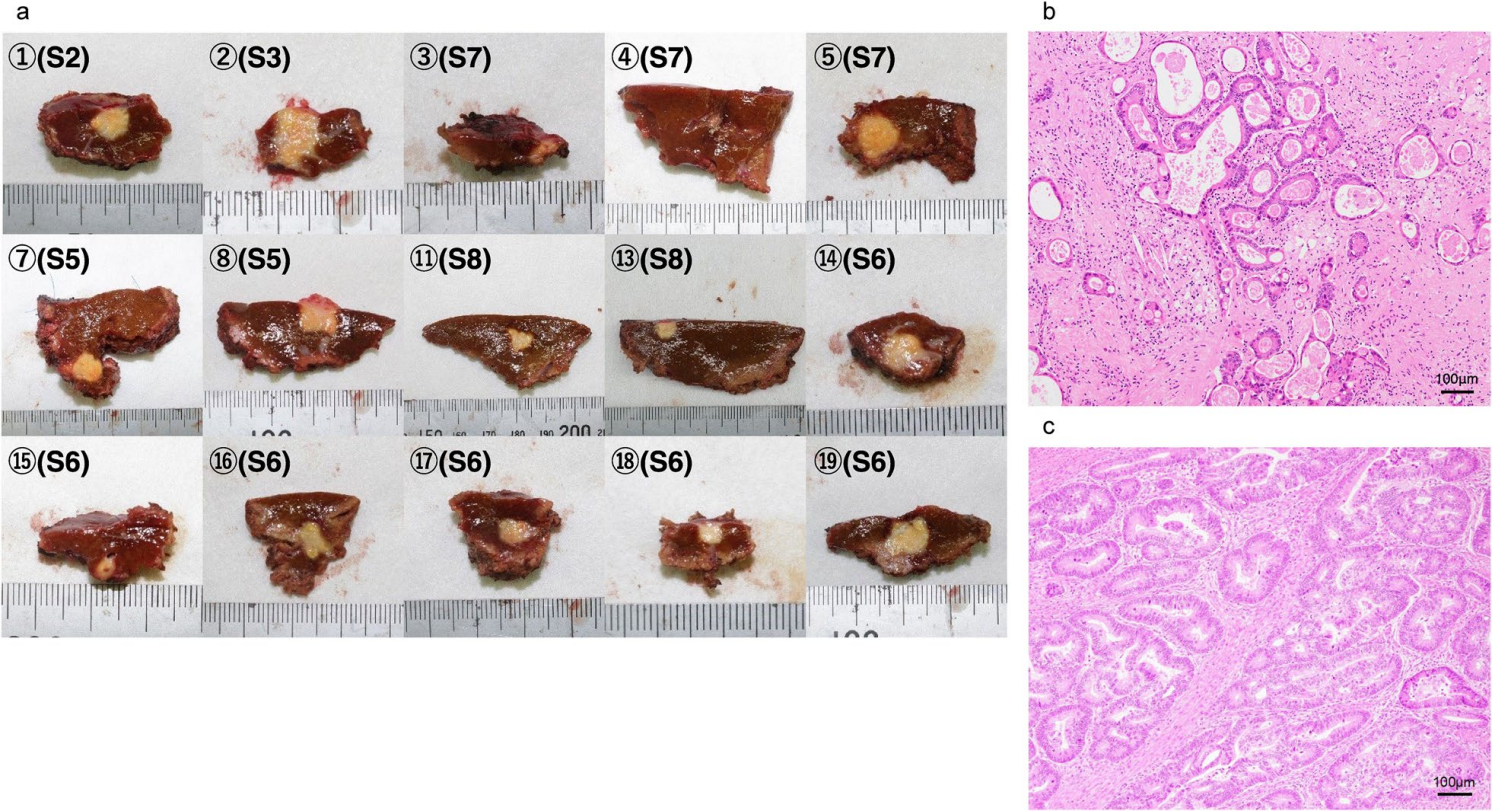
Macroscopic and histological findings of the resected specimen. **a** The fresh resected specimens. **b** Histopathological examination revealed metastatic adenocarcinoma. **c** Histopathological findings of ascending colon cancer

We considered the multiple LMs as borderline resectable. Because of the good general and biological status of the patient, bevacizumab + FOLFOXIRI was initiated to control and facilitate resection of the primary and metastatic tumors. One cycle of this regimen consisted of intravenous infusion of bevacizumab (5 mg/kg), irinotecan (165 mg/m2), oxaliplatin (85 mg/m2) concomitantly with infusion of levofolinate (200 mg/m2), followed by a 46-h continuous infusion (3200 mg/m2) of 5-fluorouracil. The regimen was delivered every 2 weeks for 12 weeks before re-evaluation of the tumor. After six cycles of bevacizumab + FOLFOXIRI, abdominal enhanced CT and magnetic resonance imaging showed shrinkage of the 19 LMs in both lobes (Fig. 1b). Colonic tumor and swollen lymph nodes had also shrunk. Partial resection of each of the 19 multiple LMs was performed. The recovery was uneventful. Postoperative pathology revealed that 16 of the 19 resected liver tissues contained viable tumors, and all surgical margins were negative (Fig. 2a,b). The maximal size of the tumors was 12 × 8 mm. The tumor regression grade was 1b.

Four weeks after surgery, the patients received four cycles of bevacizumab + FOLFIRI. After chemotherapy, serum carcinoembryonic antigen and carbohydrate antigen 19–9 levels normalized to 4.7 ng/mL and 13.4 U/mL, respectively. Left hemicolectomy with D3 lymph node dissection and colostomy closure were performed (the first R0 operation). Postoperative pathological examination revealed well to moderately differentiated adenocarcinoma (Fig. 2c), and that the depth of invasion was T3 and the tumor size was 36 × 22 mm. One of 26 lymph nodes was metastasized by carcinoma cells (#231 × 1). Tumor regression was Grade 1b. We decided not to administer adjuvant chemotherapy after surgery.

**Fig. 3:**
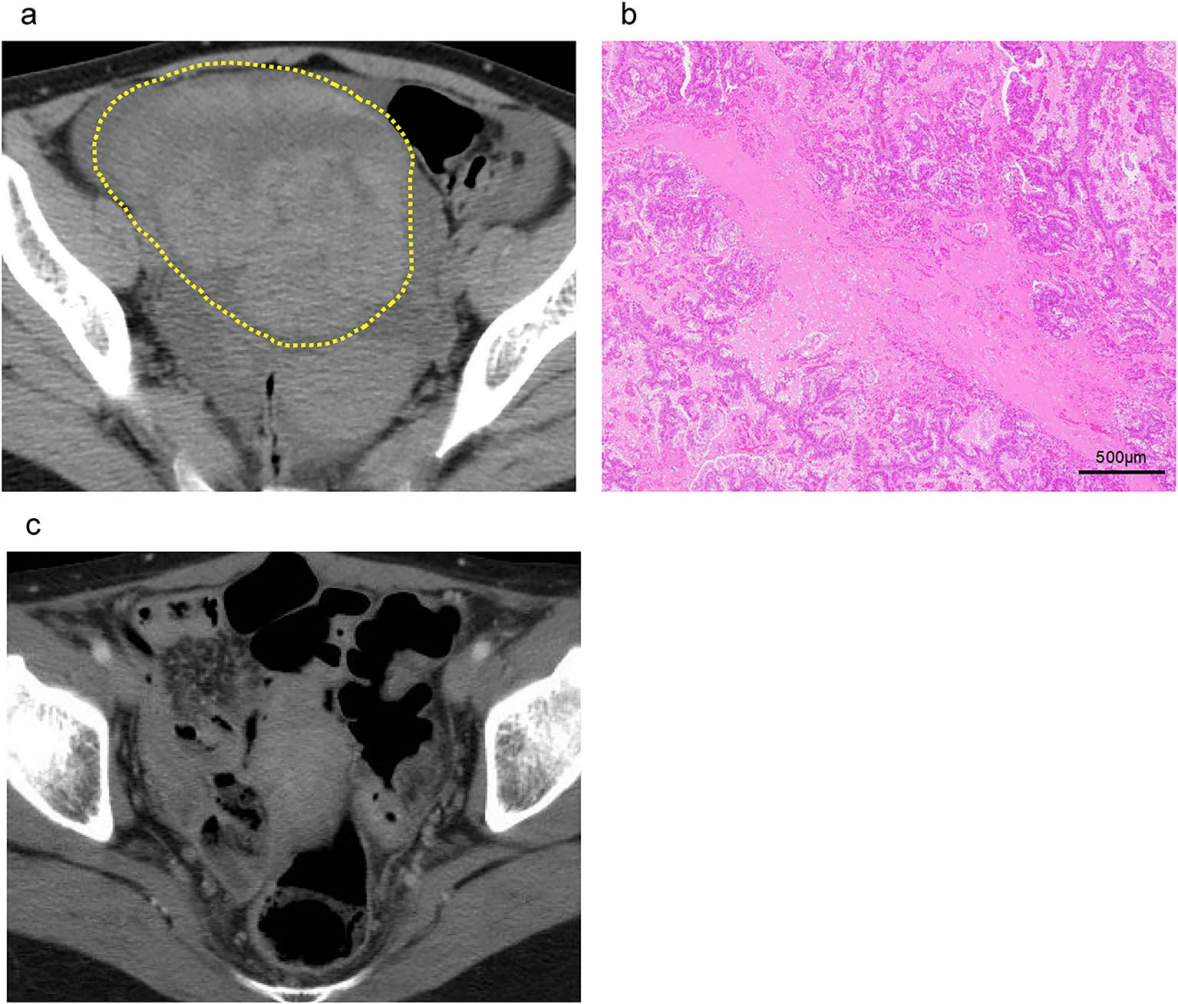
**a** Pelvic ascites fluid and ovarian tumor (yellow line). **b** Histopathological findings of the ovary tumors composed of moderately differentiated adenocarcinoma accompanied with necrosis. **c** CT image demonstrating disappearance of malignant ascites during Aflibercept + FOLFIRI treatment

Twelve weeks after colectomy, the patient experienced abdominal fullness and was hospitalized. Abdominal CT showed moderate ascites fluid and ovarian tumor measuring 10.9 × 10.1 × 7.7 cm, which indicated ovarian metastasis (Fig. 3a). We conducted bilateral oophorectomy. Histopathology revealed that the left ovarian tumor consisted of moderately differentiated adenocarcinoma growing in tubular and papillary patterns, resembling the features of the primary tumor. Furthermore, the tumor exhibited characteristics of ovarian metastasis from colorectal carcinoma, such as garland pattern and dirty necrosis (Fig. 3b) [8]. So, we diagnosed the ovary tumor as metastatic colon cancer. Intraoperative cytological examination of ascites was class V (R1 resection). After surgery, we chose aflibercept + FOLFIRI therapy every 2 weeks considering the possibility of resistance to bevacizumab; the massive ovarian metastasis with carcinomatous ascites occurred three months after the last surgery. One cycle of aflibercept + FOLFIRI regimen consisted of intravenous infusion of aflibercept (4 mg/kg) followed by irinotecan (150 mg/m2), infusion of levofolinate (200 mg/m2), and a 400 mg/m2 bolus and 46-h continuous infusion (2400 mg/m2) of 5-fluorouracil. There was no sign of recurrence or metastases for 2 years (Fig. 3c). Therefore, we decided to discontinue aflibercept + FOLFIRI therapy (total 36 cycles) and continued follow-up without chemotherapy.

**Fig. 4:**
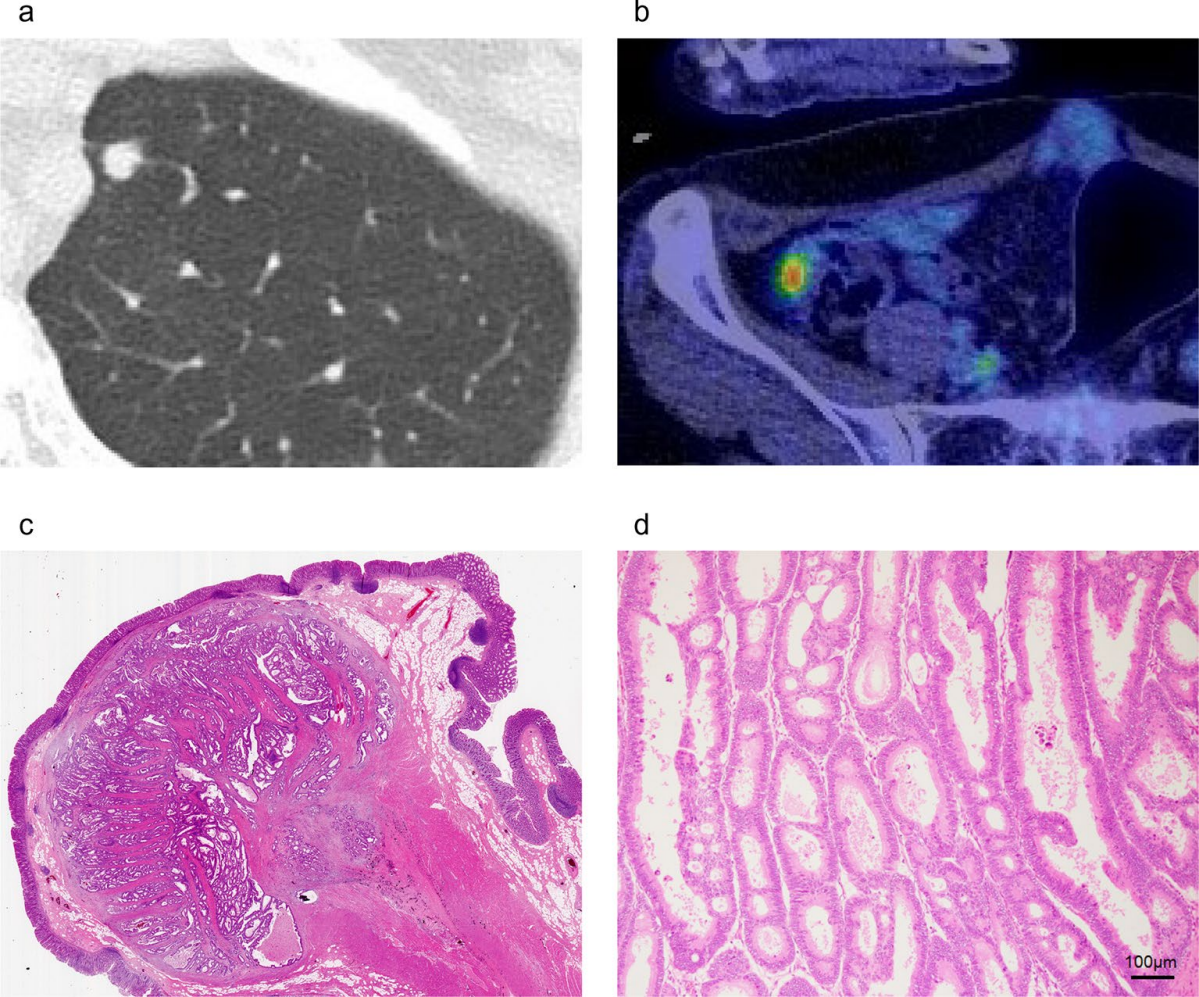
**a** One of the lung tumors. **b** Moderately intense accumulation of 18F-fluorodeoxyglucose in the cecal tumor. **c**, **d** Histopathological findings of cecal tumor. Adenocarcinoma cells occupied the submucosal to subserosa layer

Six months after the end of chemotherapy, two lung nodules were detected in the left upper and lower lobes, suggesting lung metastases (Fig. 4a). We performed thoracoscopic partial resection of the lung for both metastases (second R0 resection). Histopathology showed metastatic adenocarcinoma from colon cancer. We continued follow-up without chemotherapy after surgery.

**Fig. 5:**
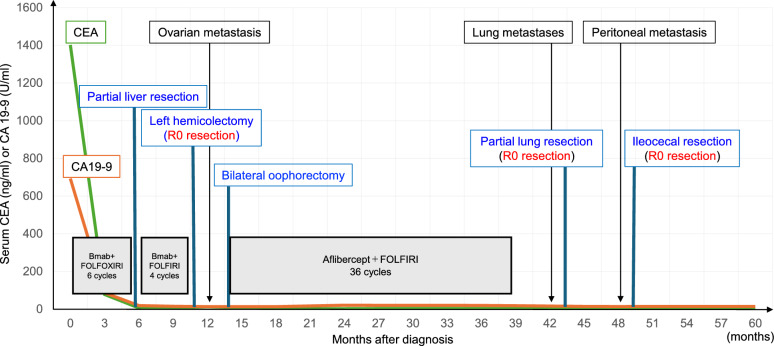
Clinical course of the patient and trends in CEA (green line) and CA19-9 (orange line) values. Bmab: bevacizumab; CEA: carcinoembryonic antigen; CA 19–9: carbohydrate antigen 19–9

Six months after surgery, a tumor was detected by CT in the cecum, and positron emission tomography/CT showed moderately intense accumulation of 18F-fluorodeoxyglucose in the tumor (Fig. 4b). Colonoscopy showed a submucosal tumor-like lesion in the cecum. From these findings, peritoneal dissemination was suspected, and ileocecal resection was performed. Intraoperative cytological examination of ascites fluid was negative for carcinoma. Histopathology showed adenocarcinoma cells occupying the area from the submucosal layer to the subserosa, which was compatible with peritoneal dissemination from primary colon cancer (Fig. 4c, d). Surgical margins were free of carcinoma cells (third R0 resection). We continued follow-up without chemotherapy after surgery. The patient did not develop any recurrence over the following 2 years and 3 months, and has survived for 6 years since the initiation of treatment (Fig. 5).

## Discussion

This was a case of RAS mutant transverse colon cancer with simultaneous multiple LMs at initial diagnosis, with long-term disease-free survival. There were two components to the strategy for achieving long-term survival.

The first was aggressive chemotherapy and radical surgery for CRLMs. CRLMs are classified into three categories: initially resectable, borderline resectable, and initially unresectable [1]. Borderline resectable tumor entails the possibility of radical resection, but is considered more challenging technically or oncologically compared with initially resectable tumor. For such patients, hepatectomy after chemotherapy is usually recommended [1]. In potentially resectable patients with conversion surgery as the goal, a regimen yielding high response rates and/or significant tumor size reduction (shrinkage) is advisable [9, 10]. In patients with RAS mutant tumor, a cytotoxic doublet plus bevacizumab or bevacizumab + FOLFOXIRI are recommended [9]. Some reports have shown that bevacizumab + FOLFOXIRI significantly improves R0 resection rate of metastatic CRC compared with other regimens [11]. Based on this evidence, we selected bevacizumab + FOLFOXIRI as first-line treatment.

There are several reports on the treatment of patients with synchronous CRLMs, including simultaneous colorectal and hepatic resection, colorectal resection followed by liver resection (primary tumor-first approach), and a reverse strategy (liver-first approach) [2, 12]. The superiority of each approach was not fully examined but some studies have indicated that the liver-first approach is associated with longer survival than the other approaches in the presence of multiple bilobar CRLMs [2, 13]. This approach maximizes the efficacy of neoadjuvant chemotherapy by allowing resection of liver metastases at the peak of response without interrupting treatment. [2]. In our case, the primary tumor was well controlled by bevacizumab + FOLFOXIRI. Control of LMs was considered important; therefore, we decided to adopt the liver-first approach. Re-evaluation by CT at 3 months after liver resection revealed no new metastases; therefore, we performed resection of the primary tumor (first R0 resection).

The second component of our strategy for achieving long-term survival was chemotherapy for the RAS mutant tumor. In particular, aflibercept + FOLFIRI therapy resulted in prolonged survival after surgery for massive ovarian metastasis. Aflibercept is an antiangiogenic agent that targets vascular endothelial growth factor (VEGF)-A, VEGF-B and placental growth factor (PlGF) [14]. Aflibercept is approved in combination with FOLFIRI in patients with metastatic CRC, who have disease progression following oxaliplatin treatment [15]. In the VELOUR trial, in patients with metastatic CRC previously treated with oxaliplatin, combination of aflibercept + FOLFIRI provided a significant survival advantage over the combination of FOLFIRI and placebo [16]. Disease control rate was 85.7% in the aflibercept + FOLFIRI group and 76.1% in the placebo + FOLFIRI group [16]. It is reported that aflibercept + FOLFIRI and bevacizumab + FOLFIRI are equally effective second-line therapies in patients with RAS mutant metastatic CRC [17]. Aflibercept + FOLFIRI was also reported to be effective in patients with KRAS mutation, who were treated previously with a combination of oxaliplatin and bevacizumab [5, 7]. Among the phase III trials evaluating the efficacy of angiogenesis inhibitors in second-line treatment for colorectal cancer, aflibercept + FOLFIRI had the highest response rate (ORR:19.8%) in the VELOUR trial [18]. In addition, in contrast to the other trials, the VELOUR eligibility criteria included patients with a predicted poor prognosis, especially those with rapid recurrence after postoperative adjuvant therapy [7]. Considering these points, although it is generally not recommended to compare results between different clinical trials, we chose aflibercept + FORFILI therapy as the most effective second-line therapy for this patient with ovarian metastasis shortly after bevacizumab + FORFILI therapy. The patient did not relapse during 2 years of aflibercept + FOLFIRI treatment but relapsed shortly after discontinuation. Aflibercept may have inhibited recurrence. The VELOUR trial's post hoc analysis revealed that patients previously treated with bevacizumab showed a significant increase in VEGF-A and PlGF in plasma compared to those who had not received prior bevacizumab. Furthermore, it was observed that aflibercept had better results than a placebo in patients with high baseline levels of VEGF-A or PlGF and no prior bevacizumab treatment [19]. These findings suggest that treatment with bevacizumab may result in elevated VEGF-A and PlGF levels, potentially leading to bevacizumab resistance, while also contributing to a favorable response to aflibercept. After surgery for lung and ileal recurrences, additional chemotherapy was not added, at the patient’s request, but it may be a treatment option in the event of future recurrence.

A multidisciplinary approach is optimal to achieve better survival of patients with metastatic CRC. While several new chemotherapeutic and biological agents have been developed, surgical resection remains the best option for achieving long-term survival in some patients with liver or lung metastases [20]. Even though the postoperative recurrence rate of distant metastases is 50–70%, repeated resection of metastases may improve the long-term survival of patients with metastatic CRC [20–22]. Hannes et al. reported that repeated hepatic resection for CRLMs can be performed with low mortality and acceptable morbidity, and survival rate after repeated resection is promising and comparable with that after initial hepatic resection [23]. Francesca et al. reported that radical oophorectomy for ovarian metastasis from colorectal cancer has a good impact on disease-free and overall survival [24]. Hishida et al. reported that repeated lung resection for lung-limited recurrence after pulmonary metastases from CRC is as favorable as initial pulmonary resection [25]. Ozawa et al. [26] and Bang et al. [27] reported that R0 resection of peritoneal metastases from CRC should be considered in selected patients. However, there are few reports of resection of peritoneal metastases.

Some studies have revealed that aggressive R0 resection does not improve the survival of CRLMs and may even worsen prognosis and quality of life in older patients or patients with low performance status. Therefore, treatment indication should be discussed in a multidisciplinary conference. In this case, we discussed the treatment in a conference and chose the curative option because the patient was young and had strong hopes for curative treatment.

## Conclusion

Long-term recurrence-free survival for CRC patients with marginally resectable metastases or repeated recurrence may be achieved by combination chemotherapy, considering the genetic background of the tumor, and aggressive R0 resection. Aflibercept may be effective in controlling metastasis of RAS mutant tumor even after treatment with bevacizumab.

## Data Availability

Data sharing is not applicable to this article as no datasets were generated or analyzed during the current study.

## Abbreviations

CRLM: Colorectal liver metastases
CRC: Colorectal cancer
CT: Computed tomography
PET-CT: Positron emission tomography/computed tomography
18F-FDG: 18F-fluorodeoxyglucose
CEA: Carcinoembryonic antigen
CA 19–9: Carbohydrate antigen19-9
